# How to Design Optimal Accelerated rTMS Protocols Capable of Promoting Therapeutically Beneficial Metaplasticity

**DOI:** 10.3389/fneur.2020.599918

**Published:** 2020-11-05

**Authors:** Alix C. Thomson, Alexander T. Sack

**Affiliations:** ^1^Department of Cognitive Neuroscience, Faculty of Psychology and Neuroscience, Maastricht University, Maastricht, Netherlands; ^2^Department of Psychiatry and Neuropsychology, School for Mental Health and Neuroscience (MHeNS), Maastricht, Netherlands; ^3^Centre for Integrative Neuroscience, Faculty of Psychology and Neuroscience, Faculty of Health, Medicine and Life Sciences, Maastricht University, Maastricht, Netherlands

**Keywords:** metaplasticity, homeostatic plasticity, hebbian plasticity, transcrancial magnetic stimulation (TMS), accelerated rTMS

## Introduction

Our brain is comprised of billions of neurons, which can connect via synapses that rely on electrical signaling and the release of chemical messengers to communicate and propagate signals through neural networks. By forming such networks, neurons are capable of monitoring previous firing activity, and using this information to adapt subsequent firing rate. This so-called activity-dependent plasticity is critical for the encoding of new information, and the tuning of (low activity) connections (1–3). The physiological mechanisms of synaptic plasticity have largely been attributed to Long-Term Potentiation (LTP) (4, 5), and Long-Term Depression (LTD) (6–8), which result from molecular processes such as receptor trafficking or synaptic scaling (3). Both LTP and LTD are induced by postsynaptic NMDA receptor activation, which lead to an influx of calcium into the postsynaptic dendrites (8–10). This triggers a complex series of intracellular signaling cascades, resulting in synaptic modifications such as AMPA receptor trafficking (11, 12). The pattern of stimuli delivered to the post synapse determines whether LTP or LTD will occur; low frequency stimulation induces LTD, whereas high frequency stimulation induces LTP (8, 13). These processes underlie much of our knowledge on the molecular mechanisms of learning and memory.

However, if the principles of Hebbian synaptic plasticity (LTP, LTD) alone were to drive the strengthening and weakening of synaptic connections, activity would, over time, be driven toward destabilization. This is because continuously firing synapses could only become stronger (driven to saturation) and unused synapses quiescent (until completely lost) (14). Consider a synapse that is strengthened by LTP; meaning the presynaptic neuron becomes more effective at depolarizing the postsynaptic neuron. With each continued stimulation, the postsynaptic neuron will be more easily depolarized, in a positive feedback loop, resulting in a hyperexcitable postsynaptic neuron. Over time, not only will the original presynaptic connection be strengthened, but other unrelated presynaptic inputs could cause a depolarization of the hyperexcitable postsynaptic neuron, resulting in unregulated synaptic transmission (15). Therefore, other mechanisms must exist, which regulate synaptic plasticity on a global network level to maintain stability of synapses and maintain specificity of neural activity (16, 17).

Metaplasticity refers to any change in the direction or degree of synaptic plasticity (ex. LTP, LTD) based on prior neural activity (18). While both synaptic and metaplasticity are dependent on previous neural activity, metaplasticity does not *directly* alter the efficacy of synaptic transmission (as LTP/LTD), but it adjusts the neurons' ability to induce LTP/LTD with *subsequent* neural activity. Metaplasticity in some sense can be considered as the plasticity of synaptic plasticity, e.g., maintaining the dynamic nature of a neuron's firing threshold, when this neuron reaches a certain firing rate (16, 18, 19). Metaplasticity works through similar synaptic modifications as LTP/LTD, such as NMDA receptor activation and modification (20), and changes in calcium signaling triggering complex signaling cascades (18). Metaplastic modifications, for example at NMDA receptors, can occur either at specific synapses or across the whole neuron, and on time scales from minutes to weeks (19). Depending on the temporal pattern and strength of previous neural activity, metaplastic mechanisms can be additive; for example promoting increased synaptic strengthening through repeated excitatory (LTP-inducing) stimulation. Metaplasticity can also be stabilizing; for example acting against subsequent synaptic strengthening when repeating excitatory (LTP-inducing) stimulation (19, 21). This stabilizing form of metaplasticity is often referred to as *homeostatic* metaplasticity, as it specifically regulates the dynamic threshold of synaptic plasticity to maintain equilibrium, or homeostasis (16, 17). We hypothesize, based on research from human and animal studies, that the timing between excitatory stimulations are what differentiate between promoting additive or homeostatic metaplasticity.

We focus on the role of metaplasticity in Transcranial Magnetic Stimulation (TMS). We describe the recent use of accelerated (repeated) stimulation protocols, both in research and clinical applications, and the molecular mechanisms required to promote either homeostatic or additive metaplastic effects. Finally, we showcase the therapeutic potential of accelerated stimulation, and hypothesize that increasing the currently practiced stimulation intervals may be more efficacious in promoting additive metaplastic effects in various clinical applications of rTMS in rehabilitation, neurology, psychiatry, and cognitive decline.

## Metaplasticity in TMS

TMS is a widespread and increasingly popular non-invasive brain stimulation technique, where electromagnetic pulses allow stimulation to pass non-invasively through the skull (22). When pulses are applied in a certain pattern, as repetitive TMS (rTMS), protocols can have lasting excitatory or inhibitory effects (23–25). Two commonly used stimulation protocols are *intermittent* Theta Burst Stimulation (iTBS), requiring only 3 min of stimulation time, resulting in a lasting increase of cortical excitability, and *continuous* Theta Burst Stimulation (cTBS), requiring only 40 s of stimulation for a lasting decrease in cortical excitability (26). The after effects of these protocols have been shown for up to 1 h following stimulation (26, 27).

While iTBS is normally an excitatory protocol, causing an increase in cortical excitability of the stimulated brain region, it has been shown that when applied twice in quick succession iTBS effects switch from excitatory to inhibitory (28). Conversely, when cTBS (an inhibitory protocol) is applied for double the normal duration, its effects switch from inhibitory to excitatory (28). Several studies have reported similar effects of repeating iTBS or cTBS stimulation protocols, with the timing between protocols being an important factor in the magnitude and direction of aftereffects (19, 29, 30). For example, using a “priming” iTBS protocol which does not induce plasticity, followed by a “test” iTBS protocol has shown that short intervals of 5 min between priming and test resulted in homeostatic-like changes in excitability, i.e., an opposite effect. Interestingly, longer breaks of 15 min resulted in an increase in MEP amplitude after the test iTBS (30). However, 15 min between priming and test iTBS/cTBS has also been shown to induce in homeostatic-like metaplastic effects (29). While the timing between repeated TBS sessions is clearly important, the optimal interval is less clear. 15 min between iTBS sessions has been shown to promote both homeostatic (29) and MEP enhancement after the second iTBS (30), while 10 min between priming and test iTBS has shown enhancement of MEP amplitude (31), but 5 and 20 min between iTBS sessions did not (32). Therefore, when 2 iTBS sessions are repeated with short (<30 min) between, conflicting effects on MEP amplitude have been reported.

“Accelerated” protocols, which consist of multiple stimulation sessions on a single day, have recently been introduced for the treatment of depression (33–37). Due to their short duration, the TBS protocols, in particular iTBS, have been promising candidates for accelerated protocols (38). Also, a large trial recently found that iTBS was not-inferior to the classical 10 Hz rTMS protocol, confirming the clinical potential of this shorter stimulation protocol to treat depression (39). Indeed, several studies have shown additional benefits for accelerated iTBS protocols in the treatment of severe, treatment resistant depression (40, 41). In the clinic, an interval of 15 min is often used between iTBS sessions, with these sessions repeated up to 5 times on a single treatment day (40, 41).

We recently conducted a study investigating the effects of accelerated iTBS over motor cortex, consisting of 5 repeated iTBS sessions in a single day. iTBS with 8- or 15-min time interval between sessions were delivered to healthy participants in a fully within subject design; where participants received 4 different conditions (accelerated iTBS with 8-min intervals, accelerated iTBS with 15-min intervals, single iTBS and sham) (42). We compared change in Motor Evoked Potential (MEP) amplitude up to 90 min following stimulation, across the stimulation conditions.

We found that there was no difference in the effects of accelerated iTBS on MEP amplitude, also when compared to sham stimulation, and thus no additive metaplasticity induced by five stimulation sessions applied successively in 8- or 15-min intervals. We argue that such intervals between iTBS protocols are likely too short to avoid processes of homeostatic plasticity. With only 8 or 15 min between sessions, homeostatic mechanisms may be working against additive metaplastic effects to maintain network stability and therefore result in a net effect of no change in excitability following these accelerated protocols (42).

## Timing-Dependent Metaplasticity

In agreement with this notion, animal studies in rats, and rat hippocampal slices have shown that a sufficiently long pause between excitatory stimulation sessions was necessary for additive (LTP) plasticity effects to occur (43–45). This may have to do with the time required for metaplasticity mechanisms, for example synapse strengthening with AMPA receptor trafficking (15).

**Figure 1:**
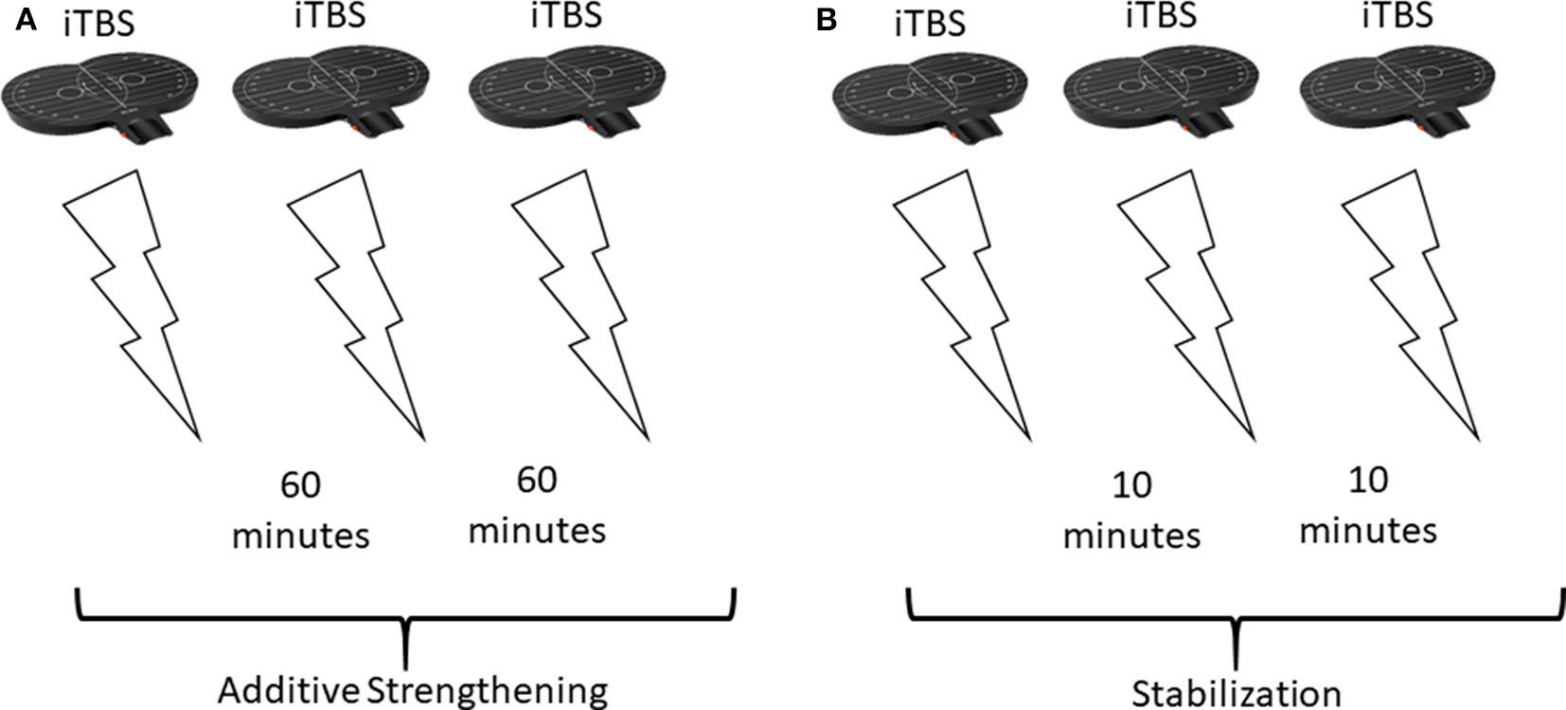
Theoretical stimulation setup and effects in response to different spacings between repeated stimulations. **(A)** Repeating excitatory (iTBS) stimulation 3 times, with 60 min between sessions, promotes additive strengthening of stimulated synapses. Overall, the repeated stimulation increases potentiation [this has been shown in animals using a different TBS protocol (43, 45)]. **(B)** Repeating the same 3 iTBS stimulations, but with only 10 min between sessions results in stabilization (homeostatic metaplasticity) and no change in overall plasticity.

It has been well-established in animal studies, that a single round of TBS (a 4-pulse burst at 100 Hz, repeated at 5 Hz for 10 bursts) is effective at inducing LTP in CA1 hippocampal pyramidal neurons (46, 47). TBS has since then been used extensively to reliably induce LTP *in vitro* (48). Interestingly, repeating this single TBS protocol with a time interval of >40 min, was capable of almost doubling the potentiation compared to the first TBS alone (43). This additional potentiation is thought to work through strengthening the smaller synapses which weren't strengthened by the first TBS protocol (43). This may have to do with the number of AMPA receptors; smaller synapses contain fewer AMPA receptors and therefore don't generate a response to trigger a depolarization following a single TBS (43). Several other studies have provided evidence for increased potentiation by spaced TBS, however the magnitude and duration of the effects depended on a series of factors such as rat strain, rat age, and the time interval. In adult Wistar rats, adult Long-Evans (LE) rats, and young LE rats, 4 h was required between TBS to induce additional potentiation (44, 45). However, in young Sprague Dawley (SD) rats, a single TBS repeated at 1-h intervals could induce further potentiation, following up to 3 repeated TBS stimulations (4 did not produce additional potentiation) (43, 45). These different studies used different stimulation intensities; Frey et al. (44) found that reducing stimulation intensity in the second stimulation was effective for promoting potentiation 4-h later, while Cao and Harris (45) and Kramár et al. (43) kept stimulation intensities constant. However, these studies consistently show that additional potentiation following repeated TBS in animal slices is possible. Enhanced, additive LTP-like plasticity may be promoted when repeating TBS with 50–60 min between sessions (43, 45). After 3 TBS protocols, spaced 60 min apart, potentiation had been raised to 150% baseline, which is about three times higher than if just one protocol was given (43, 48). This suggests that 3 TBS protocols repeated at 60 min-intervals may be effective at promoting maximal, additive metaplasticity effects (Figure 1A). If there is less time between TBS protocols, for example 10 min, homeostatic metaplasticity mechanisms may dominate, promoting a stabilizing rather than additive plasticity response (Figure 1B).

## Discussion

Activity-dependent metaplasticity is considered to be *homeostatic* if the first stimulation protocol alters the threshold for subsequent LTP/LTD in the opposite direction, thereby stabilizing (network) brain activity (49). Interestingly, this reversal of aftereffects has been shown specifically when stimulation protocols were given with a short (0–5 min) interval (28, 30), providing support for homeostatic metaplasticity mechanisms in rTMS protocols (19). While homeostatic metaplasticity mechanisms are important for stabilizing network activity, they can be counteractive when promoting plasticity effects through rTMS. In fact, when applying rTMS protocols, the explicit goal is not stabilization but promotion of additive, increased plasticity effects.

Animal studies have shown that timing is important in the molecular mechanisms underlying metaplasticity. While there is overlap between the mechanisms of additive and homeostatic metaplasticity, there are temporal differences which may differentiate between both principles at the molecular level. Based on evidence form animal models, leaving 60 min between excitatory stimulation protocols may promote additive rather than homeostatic metaplastic effects in accelerated TMS treatment protocols.

### Clinical Implications

If longer intervals between iTBS sessions are capable of promoting additive metaplasticity, as has been shown in animal studies (43) as well as improving clinical outcomes in the treatment of depression (50), longer spaced intervals between iTBS sessions will likely be beneficial for other therapeutic applications of iTBS. iTBS is increasingly being used as a treatment in a range of clinical applications such as rehabilitation, as well as neurological and psychiatric disorders. For example, to promote motor recovery after stroke (51), for managing spasticity associated with Multiple Sclerosis (MS) (52), and decreasing obsessive symptomatology associated with Obsessive Compulsive Disorder (OCD) (53), just to name a few. These protocols all must adhere to the established safety guidelines (54), and recommendations for clinical TMS use (55, 56). These include total pulse number, interval between TBS session, intensity of stimulation, and cumulative weekly applications (54). Accelerated iTBS has been successfully and safely used in the treatment of depression (38, 40, 41), with patients receiving a total of 32,400 pulses at 110% resting motor threshold, over 20 sessions (5 sessions per day, 15 min between sessions) in 4 days (41). Therefore, while following the established safety guidelines is the upmost priority, and local health authorities should always approve each stimulation protocol (54), delivering three iTBS sessions on a single day with 1 h between sessions should theoretically be safe and tolerable for most patients.

rTMS is also used as a treatment for the cognitive decline associated with neurodegenerative disorders such as dementia, and Alzheimer's Disease (AD) (57–61). However, there are ethical implications of using rTMS for cognitive enhancement, in particular in healthy participants (62). It is important to maintain the consensus ethical requirements that (1) participants/patients provide informed consent, (2) the benefit of the research outweigh the risks, and (3) there is equal distribution of burdens and benefits across patients (this is violated if a particular group of patients with different economic, physical or social conditions) (54).

Importantly, the here described principles of additive and homeostatic metaplasticity not only apply to the here discussed accelerated TMS treatments and the question of optimal time interval between its repeated stimulation sessions, but likewise can be used to explain and optimize other forms of plasticity-inducing TMS protocols such as Paired Associated-Stimulation (PAS) or paired-coil TMS (pcTMS).

In humans, neural excitability and synaptic plasticity can be probed by TMS to peripheral nerves and motor cortex (63, 64). In such a transcortical loop, timings of afferent (muscle/nerve to brain), cortical, and efferent (brain to muscle) responses can be used to quantify central motor excitability (63). For example, delivering a conditioning TMS pulse to an afferent tract (ex. the wrist), followed (10–48 ms) by stimulation of the efferent tract (motor cortex), will alter Motor Evoked Potentials (MEP's) measured from thumb flexor muscles (63). It has been shown that wrist stimulation 20–22 msec preceding motor cortex stimulation elicits a facilitated MEP, with a latency of about 1 ms, compared to MEPs given without the conditioning wrist stimulation (63). Repeating this afferent (wrist) efferent (motor cortex) stimulation, in Paired Associated Stimulation (PAS), can induce lasting effects on motor cortex excitability (64, 65), providing evidence for synaptic plasticity. Interestingly, evidence of homeostatic and additive metaplastic responses have also been recorded using PAS stimulation (66, 67). When two LTP-inducing PAS protocols were separated by 30 min, a decrease in MEP amplitude was measured, indicating a homeostatic (stabilizing) metaplastic responses (66). Similarly, LTD-inducing PAS immediately preceding a motor-learning task facilitated motor-learning (67), again providing support for homeostatic plasticity mechanisms dominating at early time points following stimulation.

Additionally, the effects of brain stimulation are not only localized to the site of stimulation, but can also spread to different areas through complex cortical networks. Similarly to PAS, this has been shown using paired-coil TMS (pcTMS), where multiple coils are used to probe different cortical areas and assess connectivity (68, 69). For example, a single TMS pulse to motor cortex can cause a depression of the MEP measured following a subsequent (6–30 ms) TMS pulse to contralateral motor cortex (70). Therefore, TMS can also be used to assess connectivity between brain areas (68). In other words, TMS stimulation can propagate to different cortical regions, having both local and remote effects on (meta) plasticity. This has valuable clinical implications, where inducing plasticity effects in a cortical network are important (69). In stroke patients for example, localized damage can disrupt connectivity and can have functional consequences (69), therefore stimulation effects should promote network plasticity, rather than localized plasticity. Similarly, in the treatment of depression, superficial stimulation uses cortical connectivity to influence deeper cortical structures, resulting in improvement of clinical symptoms (71, 72). Therefore, it is important to use TMS to strengthen connectivity, and to promote additive, metaplastic changes also on the network activity level.

With the increasing and widespread application of rTMS protocols in the clinic, it is important to optimize protocols to maximize their effects, while remaining within established safety and ethical guidelines for use in the clinic (54, 56). Single iTBS has proven promising, but accelerated iTBS at longer time intervals (60 min) between sessions could maximize clinical outcomes through additive metaplasticity, preventing homeostatic metaplasticity from stabilizing stimulation effects. Clinical efficacy of PAS and pcTMS protocols may be similarly increased by optimizing the timing between stimulations according to these principles of metaplasticity.

## Author Contributions

AS: conceptualization, writing-review and editing, supervision, and funding acquisition. AT: investigation, writing-original draft preparation, and visualization. All authors contributed to the article and approved the submitted version.

## Conflict of Interest

The authors declare that the research was conducted in the absence of any commercial or financial relationships that could be construed as a potential conflict of interest.
